# Clofarabine Experience in Children with Multi-Relapsed Acute Leukemia

**DOI:** 10.4274/tjh.2013.0297

**Published:** 2014-09-05

**Authors:** Zeynep Karakaş, Begüm Şirin Koç, Serap Karaman, Sema Anak, Ayşegül Ünüvar, Ezgi Uysalol, Ömer Devecioğlu, Leyla Ağaoğlu, Gülyüz Öztürk

**Affiliations:** 1 İstanbul University İstanbul Faculty of Medicine, Department of Pediatric Hematology, Division Oncology, İstanbul, Turkey

**Keywords:** Clofarabine, Multi-relapsed Leukemia, children, Relaps

## TO THE EDITOR

Although clofarabine is known as an effective novel agent in relapsed acute leukemia [1,2,3,4,5], determining the optimum combination with other agents and the optimum time to use remains a challenge. Clofarabine is recommended to be used after 2 protocol regimens in patients with relapsed leukemia [1]. We want to add our experience with clofarabine in children with multi-relapsed acute lymphoblastic and myeloblastic leukemia. We analyzed the data of 12 children (1-13 years old) treated with the CLOVE protocol [clofarabine (4 mg/m2), cyclophosphamide (440 mg/m2), and etoposide (100 mg/m2) for 5 days] [2,3,4] for relapsed or refractory acute leukemia between 2009 and 2013. We used this therapy in a third or more of cases of relapsed acute leukemia. Bone marrow relapses were eligible for the treatment protocol. Seven of 12 patients had acute lymphoblastic leukemia (ALL) and 5 had acute myeloid leukemia (AML) (Table 1). Patients with relapsed ALL were treated with 1 or 2 cycles of FLAG after applying the BFM-95 REZ protocol. Patients with no response were administered clofarabine. Patients with relapsed AML were treated with 2 cycles of FLAG after applying the MRC protocol. Patients without response were given clofarabine in 1 or 2 cycles. The required permission for all patients was received from the Ministry of Health.

## RESPONSE TO THE PROTOCOL THERAPY WITH CLOFARABINE

**Figure 1:**
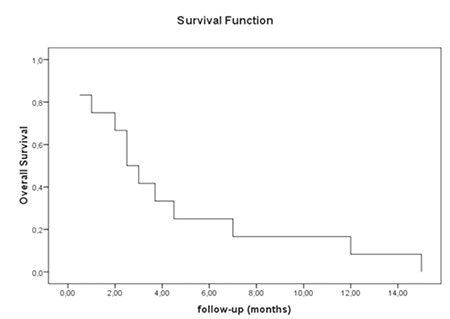
The overall survival rates from start of clofarabine treatment.

Clofarabine was effective at inducing remission in 6 patients (50%) and half of them received hematopoietic stem cell transplantation (HSCT) (Table 1). Of these 3 patients who had allogeneic HSCT after remission with the CLOVE protocol, all of them relapsed after the HSCT. One of them received a second allogeneic HSCT after the remission with the second therapy protocol with clofarabine; she relapsed again after the second HSCT. One patient had previously received allogeneic HSCT due to Ph (+) ALL; he relapsed after the HSCT and was unresponsive to the therapy protocol with clofarabine. Although clofarabine was effective to induce remission, overall survival was poor in our study. The 3-month and 12-month overall survival rates from the start of clofarabine treatment were 45.5% and 9.1%, respectively (Figure 1). All of the patients relapsed again and eventually all of them died. Even though our remission rate is higher than the rate given in an earlier French study [6] (50% vs. 37%), we agree with that study about the earlier use of clofarabine.

Our experience with the adverse effects of a combination regimen with clofarabine was similar to that of Cooper et al. [1]. Toxicities were similar to those of other intensive chemotherapy regimens. The most common adverse event was prolonged neutropenia, which caused severe infections. The median length of absolute neutrophil count recovery was 40 days. All of the patients had severe bacterial (such as gram-negative septic shock) and invasive fungal infections. Only one patient died from severe infection, while the others were successfully treated. We also observed elevated liver enzymes in most of our patients (11/12); generally, the enzymes increased to 4-fold on day 5 and decreased to normal ranges after 2 weeks. One patient with refractory AML needed pediatric intensive care due to severe hepatotoxicity and veno-occlusive disease after clofarabine therapy.

Finally, no patient was alive at the end of our study. All patients except one died from relapsed/refractory leukemia even though 4 of them had HSCT. Although we provided longer lifetimes using the therapy protocol with clofarabine for multi-relapsed acute leukemia, the patients subsequently died from uncontrolled leukemia. Therefore, we suggest that clofarabine can be used at the first relapse in leukemia with minimal residual disease determination to obtain better results. The main question that remains is whether better outcomes could be obtained with earlier clofarabine therapy.

## CONFLICT OF INTEREST STATEMENT

The authors of this paper have no conflicts of interest, including specific financial interests, relationships, and/ or affiliations relevant to the subject matter or materials included.

## Figures and Tables

**Table 1:**
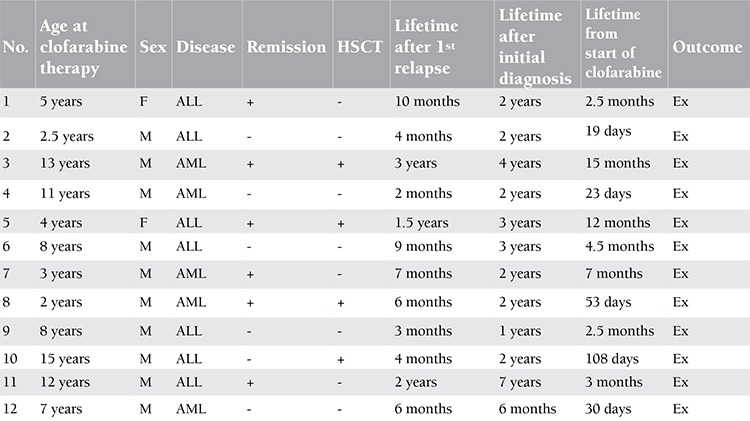
Response summary of the patients.
